# A Decade of Specialized Acute Care: Clinical Impact of an Oncology‐Hematology Emergency Room Compared to General Emergency Services

**DOI:** 10.1002/cam4.71377

**Published:** 2025-11-21

**Authors:** Norma Malavasi, Leonardo Ferrara, Lorenza Di Marco, Alessia Saviola, Raffaella Postiglione, Flavia Cantile, Gloria Acquaviva, Laura Galassi, Andrea Bergonzini, Claudia Fiorani, Laura Scarabelli, Massimo Dominici, Mario Luppi, Giuseppe Longo

**Affiliations:** ^1^ Oncological Medicine Unit and Medical Oncology Unit, Department of Oncology and Haematology, University Hospital of Modena University of Modena and Reggio Emilia Modena Italy; ^2^ Department of Oncology and Hematology, Oncology Unit, University Hospital of Modena and Reggio Emilia University of Modena and Reggio Emilia Modena Italy; ^3^ Hematology Unit, Azienda Ospedaliero‐Universitaria, Department of Medical and Surgical Sciences, Section of Hematology University of Modena and Reggio Emilia Modena Italy

## Abstract

**Background:**

The management of severe complications arising from newly diagnosed or existing cancer, as well as acute therapy‐related side effects, is traditionally provided by General Emergen‐cy Departments (GED). To address the specific needs of oncologic and hematologic patients, the University Hospital of Modena established a dedicated Oncology‐Hematology Emergency Room (OHER) in 2001 as an integral part of the Oncology and Hematology Department.

**Aim and Methods:**

This study aims to analyze the clinical characteristics and outcomes of cancer patients admitted to OHER between 2009 and 2019, including hospitalization rates, and compare them with those of cancer patients admitted to GED over the same period. A dedicated electronic tool was developed to process medical records. The OHER staff includes oncologists and hematol‐ogists trained in internal medicine, supported by a specialized nurse who is available during daytime workdays.

**Result:**

A total of 28.680 OHER admissions were recorded, involving 11.239 patients. Among them, 5.326 (47%) had a single visit, while 165 (0.6%) died during their monitoring in OHER. Admis‐sions peaked in January (10%; 2,900 visits) and were lowest in December (6.8%; 1,952 visits). The busiest day was Monday (6,926 visits), at least in terms of hospitalization and mortality rates. At our Center, OHER serves as the primary point of reference for oncology and hematological patients who present with unexpected clinical deterioration.

**Conclusion:**

Our analysis aims to provide clinical data from a dedicated oncology and hematology emergency room, comparing them with those observed in the general emergency room. In our ex‐perience, the OHER enhances the delivery of patient‐centered oncology care and the quality of comprehensive oncology and hematology care through its innovative integration of various clinical and organizational aspects.

## 1 Introduction

Cancer is the second leading cause of death worldwide, accounting for an estimated 9.6 million deaths in 2018, and remains a major contributor to global morbidity and mortality [1]. Advances in oncologic treatments have transformed cancer into a chronic condition for many patients rather than an acutely fatal disease. However, the rapid expansion of these therapies has led to a growing demand for emergency care, often extending into end‐of‐life palliative management [2].

Currently, oncologic, and hematologic emergencies are primarily managed in General Emergency Departments (GED). A systematic review of GED utilization by cancer patients found that their visit rates significantly exceeded those of the general population [3].

A population‐based study in North Carolina reported that among 358.283 estimated cancer survivors, 27,644 (7.7%) visited a GED 37,760 times (an average of 1.4 visits per person). The most common reasons for these visits included pain, respiratory complications, and gastrointestinal issues [4]. Furthermore, hospitalization followed in most cases (62.3%) [4].

Similarly, a 6‐month study in Turkey analyzing all GED visits by cancer patients found that the most frequent presenting symptoms were pain (24%), shortness of breath (17%), nausea and vomiting (14%), and fever (13%), with fatigue, diarrhea, and malaise each accounting for less than 10% [5].

Bos et al., in a nationwide study in the Netherlands, using data from the Dutch National Intensive Care Evaluation (NICE) registry (2006–2011), identified 36,860 cancer patients requiring intensive care admission, of whom 2374 (6.4%) were initially evaluated in a GED [6].

Of the estimated 696 million GED visits between 2006 and 2012 in the United States, 29.5 million (4.2%) were related to cancer complications [7]. The most frequently reported conditions included pneumonia (4.5%), non‐specific chest pain (3.7%), urinary tract infections (3.2%), and septicemia (3.1%). Lash et al. emphasized the need to determine whether cancer patients sought emergency care due to limited access to oncology clinics. They highlighted the importance of GED staff receiving specialized training to optimize cancer‐related emergency care [8].

Despite the growing need for specialized acute care, few emergency and intensive care services data are exclusively dedicated to oncologic and hematologic patients. Recognizing this gap, our institution established a dedicated Emergency Room within the Department of Oncology and Hematology (OHD). This is one of the first emergency care services for oncologic and hematologic patients in Italy to employ a short‐term monitoring model exclusively dedicated to these patients.

Our analysis characterizes patient demographics and visit patterns by day and month, providing data on clinical outcomes, particularly regarding improved quality of life and survival benefits for hematologic‐oncology patients.

## 2 Patients and Methods

The Oncology Hematology Emergency Room (OHER) at the University Hospital of Modena has been operational since 2001, providing specialized emergency care services dedicated to patients with oncology and hematology conditions. It functions as an integral part of the OHD, distinguishing itself from the GED in several key aspects:

1. Patient population: only oncologic and hematologic diseases are admitted, regardless of disease stage, from suspected malignancy to palliative care.
2. Specialized medical team: the OHER staff consists of oncologists and hematologists trained in internal medicine, ensuring high‐level expertise.
3. Seamless integration with OHD: The OHER is closely connected to the department, facilitating continuity of care.

In addition to patients with malignancies, evaluable hematologic patients include those with non‐malignant hematologic disorders, such as coagulopathies and autoimmune diseases. The OHER serves various clinical needs, ranging from the rapid diagnosis of suspected malignancies or other hematologic conditions to managing acute complications and end‐of‐life care.

All living patients, whose data were analyzed, agreed to participate in the study “Retrospective study on the percentages of hospitalizations of onco‐hematological patients who access the Emergency Department of the Policlinico University Hospital of Modena and the onco‐hematological emergency clinic from the Emergency Department/Emergency with attached Short Observation” and signed the related informed consent.

### 2.1 Operational Framework

The OHER operates from 8:00 am to 6:00 pm, Monday through Friday, and from 8:00 am to 12:00 am on Saturday, but is unavailable at night, on Sundays, or on holidays. Its team consists of oncologists or hematologists working in the OHD, as well as a dedicated nurse.

Upon arrival, whether through self‐admission or via emergency ambulance, patients are triaged and stratified according to clinical severity. The only exception concerns cases of suspected acute cerebrovascular emergencies, identified in advance through telephone communication with ambulance teams; these patients are directly referred to a specialized neurological emergency center in the area.

Patients with a known oncological diagnosis and already under the care of our Center's Day‐Hospital undergo immediate laboratory tests and diagnostic imaging, allowing for the prompt initiation of targeted assessments and treatments.

Individuals presenting with acute conditions are admitted to a short‐term intensive observation unit, where they receive primary care, specialist consultations as needed, and stabilization measures. This observation period is used to determine whether hospitalization or safe discharge is the most appropriate course of action.

Based on clinical evolution, patients are either admitted to the Oncology or Medical Oncology departments of our Center or discharged with a structured follow‐up plan through our Day‐Hospital services.

### 2.2 Electronic Data Management

A computerized system collects and integrates disease history, prior hospitalizations, scheduled and urgent evaluations, radiological and laboratory findings, specialist consultations, and administered treatments, including clinical trial drugs. These data are routinely recorded for clinical management and administrative purposes and made readily available to all healthcare providers. The electronic health record (EHR) platform consolidates all variables, allowing efficient decision‐making and optimized patient care strategies.

### 2.3 Data Collection and Analysis

- *Primary analysis*: Data were retrospectively extracted using specific queries to analyze patient diagnoses, visit frequency, reasons for OHER access, and timing of visits (in hours). Hospitalization rates from the OHER were also determined.
- *Comparative analysis*: A separate GED database covering the same period was reviewed to identify visits by patients with oncology and hematology conditions. This enabled us to calculate GED hospitalization rates, with particular attention to cases occurring at night or on public holidays, when the OHER was not operational. It is essential to emphasize that an oncology consultant from our Center is available 24/7 to support the GED whenever the OHER is unavailable.
- *Sepsis‐related mortality*: All hospitalized patients in the OHD with a sepsis diagnosis were identified, and the percentage of sepsis‐related deaths was calculated.

## 3 Results

Between January 1, 2009, and December 31, 2019, 28,680 admissions were recorded at the OHER. Among the 11,239 evaluated patients (median age: 65 years; 49.9% women, 50.1% men), 5326 (47%) had a single visit, while the remaining 53% had multiple visits. The two patients with the most frequent returns, diagnosed with hemophilia, had 51 and 50 visits, respectively (Table 1).

**TABLE 1:** Recurrence of visits in OHER.

No. of accesses	No. of patients
31–50	14
19–30	28
11–18	120
1–10	11.017
Total	11,239

The median overall survival after the first OHER visit was 40 months, with a significant difference between sexes:

- 69 months for women.
- 26 months for men.

The observed difference in median overall survival between sexes (69 months for women vs. 26 months for men) does not appear to be related to disease stage at the time of OHER presentation. Instead, it more likely reflects the underlying epidemiology of cancer types within the respective populations. In our cohort, women were predominantly affected by malignancies such as breast cancer, which generally carry a more favorable long‐term prognosis. In contrast, men were more frequently affected by tumors with poorer expected outcomes (e.g., lung cancer). For this reason, the survival gap is better explained by the distribution of cancer types rather than by stage at first OHER access.

Long‐term survival analysis showed that:

- 43% of patients were alive 5 years after their first OHER visit.
- 30% of patients were alive 10 years after their first OHER visit.

### 3.1 Primary Diagnoses

The most common diagnoses among OHER's patients, including both oncologic and non‐malignant hematologic conditions, were:

- Digestive tract cancers: 6909 cases (24%)
- Lung cancer: 4745 cases (16.5%)
- Acute and chronic leukemia: 2880 cases (10%)
- Lymphoma: 2613 cases (9%)
- Colorectal cancer: 2437 cases (8.4%)
- Urological cancers: 2349 cases (8%)
- Breast cancer: 2078 cases (7.2%)
- Multiple myeloma: 1379 cases (5%)
- Head and neck cancers: 882 cases (4%)
- Other hematologic disorders: 676 cases (2.3%)
- Sarcoma: 494 cases (1.7%)
- Melanoma: 402 cases (1.4%)
- Thrombotic thrombocytopenic purpura: 77 cases (0.2%)
- Other diagnoses: 750 cases (2.3%)

### 3.2 Survival by Cancer Type

Among the two most frequent malignancies, median survival following the first OHER visit was:

- Digestive tract cancers: 21 months, with 27% of patients surviving at least 5 years
- Lung cancer: 11 months, with 12% of patients surviving at least 5 years

### 3.3 Reasons for OHER Access

Chief complaints leading to OHER visits were categorized into major clinical groups (Table 2):

- Disease progression: 32%
- Pain: 13%
- Therapy‐related toxicities: 9%
- Suspected malignancy: 6%
- Deferrable conditions: 7%
- Other reasons: 21% (including infections and pulmonary embolism)

**TABLE 2:** A most common reason for visiting OHER.

Most common cause of access	No. (%)
Disease worsening progression	9120 (32)
Pain	3700 (13)
Not cancer‐related problems	3300 (12)
Therapies related toxicities	2566 (9)
Deferrable reasons	2086 (7)
Suspected tumors	1690 (6)
Others	6128 (21)
Total	28,680

### 3.4 Temporal Patterns of OHER Visits

The distribution of OHER visits varied by month and day of the week (Tables 3 and 4):

- The highest number of visits occurred in January (10.1%), while December had the lowest (6.8%). December is the month with the lowest number of accesses, which is expected because the OHER is not available on holidays. According to company instructions, patients access the GER directly.
- Monday was the busiest day (22% of visits), whereas Wednesday had the fewest visits (16.7%). The time between access to OHER and the potential decision to discharge or hospitalize is 60–120 min.

**TABLE 3:** Number of accesses per month in OHER.

Months	No. of access (%)
1	2900 (10.1)
2	2440 (8.5)
3	2768 (9.6)
4	2400 (8.3)
5	2448 (8.5)
6	2336 (8.1)
7	2412 (8.4)
8	2540 (8.8)
9	2276 (8)
10	2196 (7.7)
11	2012 (7)
12	1952 (6.8)
Total	28,680

**TABLE 4:** Percentage of accesses on weekdays in OHER.

Weekdays	No. (%)
Monday	6296 (22)
Thursday	5260 (18.3)
Wednesday	4796 (16.7)
Thursday	4900 (17)
Friday	4940 (17.2)
Saturday	2488 (8.6)
Total	28,680

### 3.5 Hospitalization and Mortality Rates

Despite the high number of OHER admissions, only a minority resulted in hospitalization:

- 6781 patients (23%) were admitted to the Oncology‐Hematology Department (OHD).
- 1710 patients (5.8%) were hospitalized in other hospital units (Table 5).

**TABLE 5:** Hospitalization from OHER and GED.

	OHER *N* (%)	GED *N* (%)
Total accesses	28,680	10,246
Hospitalizations	6781 (23%)	5961 (58%)
Single access	5326 (47%)	—
Deaths during observation	165 (0.6%)	—

A total of 165 patients (0.6%) died during their monitoring in the OHER.

### 3.6 GED Utilization During OHER Closures

During OHER non‐operational hours (nights and holidays), 10,246 oncologic and hematologic patients sought care at the GED, representing 1.4% of all GED visits during the study period.

Among these patients:

- 5961 (58%) required hospitalization (Table 2).
- We have this data individually because patients requiring hospitalization from the GED during OHER closing hours are, in most cases, admitted to our inpatient department or subsequently transferred from other hospital departments.

### 3.7 In‐Hospital Mortality and Sepsis Outcomes

- The average length of hospital stays for patients admitted to the OHD was 10 days.
- Among the 1529 patients hospitalized for sepsis, 410 (26.8%) died.
- In contrast, no deaths were reported among the 77 patients diagnosed with thrombotic thrombocytopenic purpura (TTP), a medical emergency where early diagnosis and prompt treatment can significantly improve prognosis.

## 4 Discussion

Our report documented the use of OHER by oncologic and hematologic patients during a 10‐year study period, with assessments split by month and day of the week. The data included diagnoses, reasons for visits, the impact on sepsis management outcomes, and the percentage of hospitalizations compared with those of GED.

Following other reports, patients affected by digestive tract and lung cancer have been identified as the most frequently admitted [9]. They represent a subgroup requiring higher care intensity and more articulated assistance organization. The clinical competence of an adequately trained medical team is necessary to improve many conditions leading to OHER, including the management of invasive procedures (such as paracentesis or thoracentesis) and nursing experience in supporting all medical device outcomes to relieve abdominal or respiratory suffering, thereby avoiding the need for recovery from these symptoms. A trained staff in these activities allows us to discharge patients from the service more efficiently and send them to a regular care service.

Most patients (57%) had repeated accesses; the most frequent users (51 and 50 visits) were affected by hemophilia. Medical team experience with rare hematologic diseases, such as coagulopathies, contributes to reducing hospitalizations when conditions are stabilized. This is also a result of a direct functional connection to specialist services that regularly follow these patients, supporting the administration of specialized therapies during the OHER observation regimen.

Most patients went to the OHER due to symptoms related to disease progression (32%), pain (13%), or treatment‐induced toxicities (12%). Although pain is a common issue that may indicate disease progression [10, 11], it may also represent a failure to manage it effectively. The presence of oncologists and/or hematologists trained in pain management enabled us to find solutions for many patients by adjusting their home analgesic drug schedules. Only in this situation can OHER staff decide to refer patients to ordinary clinical services or home care assistance. Clinical expertise regarding various analgesic medications and accurate pain assessment using appropriate scoring systems is essential for evaluating this frequent reason for visits [12].

The availability of detailed reports about chemotherapies (dosage, date of administration, types of treatments, and typical toxicity) from consulting medical informatics records allows clinicians in OHER to better and more quickly recognize drug‐related side effects. As 15% of patients in the department are enrolled in clinical trials with unregistered drugs, experimental protocols can be accessed through informatics tools to identify side effects related to the study drug accurately. The investigator's brochure for ongoing clinical trials within the department is always available, which may not be possible in a GED setting.

In our experience, electronic medical records are an essential tool for understanding various aspects of a patient's history, which differentiates a general emergency department from an Oncology/hematology emergency service.

The OHER's operational coordination with other hospital services has facilitated the transition of patients from short‐term observation. In fact, each patient is discharged from the OHER only after the acute issue has been resolved and the subsequent treatment plan and appointments have been finalized with the Day Service clinical team, which is following the patient.

Likewise, active communication between OHER staff and home care services encourages patients with previously established home palliative care to return home. Seamless support between the various teams is a crucial aspect of OHER's organization, demonstrating the high level of patient satisfaction among those who do not feel isolated.

These reasons may explain the lower percentage of hospitalizations in OHER compared to GED: 6781 (23%) were for OHD, while 1710 (5.8%) were for other extra‐departmental units out of 28,680 delivered visits. During the same period, 5961 out of 10,246 patients admitted to GED (58%) required hospitalization, consistent with the literature [5]. The specific expertise in onco‐hematological diseases among the personnel at OHER, combined with the availability of complete clinical histories, may help prevent unnecessary hospitalizations.

The average hospital stay for patients in the Onco‐Hematology Internal Medicine Unit was 10 days. Although it is challenging to accurately quantify the number of hospitalization days saved by this care organization, OHER presumably led to savings of 81,430 days and a hypothetical financial benefit.

A crucial example of the potential benefits of such emergency care in an Oncology/Hematology Department is the management of sepsis. Compared to the overall population, cancer patients are nearly three times more likely to be hospitalized with severe sepsis [13]. Deaths from sepsis account for almost 10% of all cancer deaths [14]. Furthermore, the literature indicates that the average hospital mortality rate for these cancer patients is 37.8%; however, in our experience, the average hospital mortality for sepsis has been 26.8%, which is lower than reported. This may suggest that managing these patients with specifically skilled personnel can have a significant impact on clinical outcomes [15]. Prompt detection of suspected high‐risk infection cases [16].

A lower mortality rate for sepsis in our patients can be an essential indicator of the quality of care. The short observation period is a relevant aspect for quicker specialized treatments.

Seven percent of hospitalizations are due to medical conditions related to underlying conditions. This may be because patients turn to this service for its speed and ease of access. This is further supported by the typical activity recorded in January and on Mondays, which are the busiest periods. The similar average length of stay in GED (10.7 days) and OHER (10 days) suggests that patients admitted during the holidays do not exhibit different clinical characteristics; however, they tend to avoid GED and prefer to delay assessments by OHER staff since it operates within the same care unit.

Although patients need to be better trained in using the OHER for non‐acute needs, these data confirm that the unit represents a solid point of reference for all patients cared for by the OHD. An internal patient survey confirms that over 90% of patients who have visited the OHER rate the care provided by the clinical staff (physicians and nurses) as very adequate. In other cases (5.6%), the OHER serves as a point of reference for general practitioners, who can directly refer suspected cancers and promptly initiate the diagnostic and therapeutic path.

Finally, another defining feature of the OHER is its logistical advantage. In most centers, patients with high‐risk infections, such as immunocompromised individuals, often need to be referred to the emergency department due to the lack of dedicated care units. Furthermore, the general emergency department is often not an optimal environment for patients with terminal illnesses. In our experience, 165 patients (0.6%) died during OHER observation in dedicated rooms in the Oncology Department, which provided adequate palliative care, privacy, and support for family members, as well as improved end‐of‐life management.

The OHER model, if implemented in facilities managing onco‐hematological diseases, could improve clinical outcomes, and its presence in hospitals could be a significant indicator of the quality of care for oncology and hematological patients.

## 5 Conclusions

This is the first analysis to provide clinical data from a dedicated oncology and hematological emergency department, comparing them with data observed in the emergency department. In our experience, the OHER enhances the delivery of patient‐centered oncology care and the quality of comprehensive care in oncology and hematology, thanks to its innovative integration of numerous clinical and organizational aspects. The OHER can represent a fundamental point of reference for the management of oncology and hematological diseases. Based on these results, these data should be validated against the experience of other centers and compared with a more in‐depth analysis of GED data. Ultimately, the OHER service could, in the future, become an integral part of the oncology–hematological care pathway, enabling improved patient management.

## Author Contributions

All authors had full access to the data in the study and take responsibility for the integrity of the data and the accuracy of the data analysis. Conceptualization NM, LF, G.L. writing original draft preparation NM, LF, L.D.M.; writing review and editing NM, LF, L.D.M., AS, RP, FC, GA, LG, AB, CF, LS, MD, ML, and G.L. All authors read and agreed to the published version of the manuscript.

## Disclosure

Non‐commercial sponsor: Azienda Ospedaliero‐Universitaria Policlinico di Modena. Associazione: Angela Serra per la Ricerca sul Cancro, Modena, Italy.

Investigator: Dott.ssa Norma Malavasi.

Clinical center: U.O. Medicina Oncologica.

Ethics committee: Comitato Etico dell'Area Vasta Emilia Nord (AVEN).

Approval date: 04/12/2018.

This study is a retrospective and observational investigation based on routinely collected clinical data. No experimental interventions are planned, and all data will be collected and processed by applicable privacy laws and regulations, including the EU General Data Protection Regulation (GDPR) 2016/679, the Italian Data Protection Code, and relevant guidelines for the use of personal data for scientific research purposes.

The protocol (Prot. AOU 0031695718 del: 10/12/2018) will be submitted for review and approval to the Comitato Etico dell'Area Vasta Emilia Nord of the Coordinating Center.

For deceased patients or those lost to follow‐up, clinical data will be processed without informed consent by the provisions of the Italian Data Protection Authority's Authorizations, including:

- *General Authorization for the Processing of Personal Data for Scientific Research Purposes* (1 March 2012);
- *Authorization No. 9/2016*, as extended by *Provision No. 424/2018*;
- *Ethical Rules for Processing Data for Statistical or Scientific Research Purposes* (9 May 2024), under Articles 2‐quater and 106 of the Privacy Code.

## Ethics Statement

Ethics approval has been obtained by Comitato Etico dell'Area Vasta Emilia Nord. The Ethics Committee was established by AUSLRE resolution 2017/0373 of 28/12/2017 and subsequent amendments and operates in compliance with the Ministerial Decree of 12/05/2006 and subsequent amendments “Minimum requirements for the establishment, organization and functioning of Ethics Committees for clinical trials of medicinal products” and the Ministerial Decree of 08/02/2013 and subsequent amendments “Criteria for the composition and functioning of Ethics Committees”. We confirmed that all methods followed the relevant guidelines and the Declaration of Helsinki. 1002/2018/OSS*/AOUMO—“Studio retrospettivo sulle percentuali di ricoveri dei pazienti onco‐ematologici che accedono al Pronto Soccorso dell'Azienda Ospedaliero‐Universitaria Policlinico di Modena e all'ambulatorio Affido da Pronto Soccorso/Urgenze con annessa Osservazione Breve”.

## Consent

The authors have nothing to report.

## Conflicts of Interest

The authors declare no conflicts of interest.

## Data Availability

The data that support the findings of this study are available from the corresponding author upon reasonable request.
